# Progress of Pharmacology and Therapeutics

**Published:** 1885-09

**Authors:** 


THE PROGRESS OF PHARMACOLOGY AND THERAPEUTICS.

Such was the subject to which Professor Fraser, the President of the Section of Pharmacology and Therapeutics at the annual meeting of the British Medical Association, addressed himself in opening his Section. Few men could be found better adapted to descant upon the renaissance of pharmacology, or to point out the rational delimitation of that science. The peculiarity of the position of the Section, and its recent growth out of the older Section of Medicine, may seem to some minds to need an apologist, or, it may be, an advocate who would clearly indicate how the new Section not only has long been needed, but presents earnest of a most important and valuable future. Certainly Professor Fraser's words amply prove the positions in question. "Medicine," said the President, "is a progressive subject," and with this progress is involved its inevitable division into departments, among which, that devoted to pharmacology and therapeutics has asserted its claim to individual recognition among the sections. That such a claim has been preferred, and has received a tardy acceptance, seems incident to the very nature of the subjects. This view also is confirmed when we glance at the history of pharmacology. As is pointed out in the address, there are two ways of learning therapeutics; the one by clinical study, the other by pharmacological research. The clinical study involved too many and complex issues ever to lead to definite, at least to scientific results; so that, until resort was had to direct experiment upon the normal organism, and physicians learned therefrom what where the physiological action of drugs, no sure advance could be obtained in therapeutics. The older "systems of medicine" teem with what we at the present day regard as rank absurdities ; the remedies were less studied than were the general systems of treatment, while the systems themselves were based upon pure speculation, without any attempt being made to bring the matter to the test of actual experiment Bichât and Magendie recognized the necessity for ascertaining the action of remedies by experiments, and thus founded the science of pharmacology. But the practical business of curing disease must be furthered by such research, or the hold of pharmacology upon so pre-eminently practical a profession as that of medicine would be ephemeral, even to the vanishing point. Here Professor Fraser entertains no hesitating position: "precision," he assures is thus attained in administration, errors in selection (of remedies) will be avoided * * * successful applications will be possible where the pathological changes are of a simple kind.

A further development of this important address, is the stress laid upon the importance of recognizing and studying a pathological physiology. Morbid anatomy, so closely and zealously pursued, has replaced, for some, inquiry into the physiology of perverted function, of processes originating out of conditions nonexistent in the normal organ, or accruing upon actual new development of tissue. Save in so far as semeiology busies itself with the actual results of morbid processes, some manuals, and many clinical lectures, take little cognizance of such physiological problems. Pharmacology, which in one way conducts its researches by exciting departures from the physiological standard through the administration of drugs, naturally attaches especial importance to the study of the phenomena of the living organisms during disease. Through the successful prosecution of such a study, moreover, can therapeutics alone hope to treat disease. Without depreciating the older method, namely, that of administering a remedy, and observing its effect, it must be admitted that researches into pharmacological domains present far greater opportunities of attaining to the exactness and precision to which Professor Fraser alludes. He sees no reason why the physician of the future should not use his agents with the same certainty and delicate accuracy which characterizes the operations of the engineer. We heartily coincide in the wish for so happy a consummation, and believe that the institution of the Section of Pharmacology and Therapeutics, especially when its deliberations are presided over and directed by practical pharmacologists like Professor Fraser, will do much in promoting so desirable an end.

Undoubtedly, the progress of this department of medicine is at present hampered and crippled. It is pointed out, in the address, that the average student receives no instruction in the research-methods of pharmacology, and even, in many instances, has inculcated into his mind a certain depreciation of the scientific side of therapeutics. Materia medica and pharmacy usurp the place of pharmacology and therapeutics. Students who know nothing of the physiology of disease are, in their first or second year, doomed to "get up" certain cut and dried facts concerning plants and minerals; and upon these facts, added to an arbitrary posological table, they are examined. If they pass they are presumably cognizant of therapeutics, and, so far, apt at treating disease. When they advance a few steps further, they accumulate "tips" as to treatment, and their course is run. Such a picture will not, of course, be taken as applying universally; many of the teachers in the schools are as thoroughgoing pharmacologists as is Professor Fraser himself, and their lectures abound with purely experimental results. But, even in their case, almost insuperable difficulties are offered to any practical demonstrations of the facts they teach. Laboratories, in which pharmaceutical research can be undertaken, are in England conspicuous by their absence; so that workers in this field must either curtail their efforts within the narrowest limits, or leave their practices to study at a foreign university. That the subjects of pharmacology and therapeutics should occupy a much later period in the student's curriculum than it does at present, no one conversant with teaching will gainsay, and Professor Fraser suggests the third session. Probably, it will be long before such a change be instituted—the more so, when we remember that the selection of teachers in our London schools is only too often made rather on account of the individual than because he knows anything of his subject. Pharmacology has even now extended its ramifications so widely, that only a special and prolonged study of its facts and its methods can render a man in any way competent to teach it to a class. In despite of this, we find lecturers who have neither shown taste or power for prosecuting research, and who pretend to no erudition save a fair knowledge of the *British Pharmacopœia.*

When Professor Fraser's paper has been widely read and carefully considered, we may hope that this far-sighted view of pharmacology will receive a more universal acceptance than has hitherto fallen to its lot, while the requirements for its progress being recognized will be met. There is no doubt that the influence of such an address cannot but be highly salutary. Although it may be urged that but little is said about the progress of pharmacology and therapeutics, yet what is emphasized is really far more to the point. To indicate that pharmacology is a science, and capable of enlisting in its service scientific methods, is a great step.

One other point upon which Professor Fraser touched with a light hand is the inclination there is for men unfamiliar with the new Section to betake themselves and their papers, although dealing with pure therapeutics, to the Section of Medicine. Such an obvious mistake should be remedied by the secretaries when they receive the papers, as much valuable time and material is wholly lost through introducing discussions into wrong Sections.*—*British Medical Journal.*

* Whilst many of the complaints that have of late years been made of the defects of teaching, and the limited requirements of many American colleges for a degree in medicine, have not been without good cause, it seems apparent, from the foregoing extract from *The British Medical Journal*, that such defects are not limited to the American side of the Atlantic ocean, but that other countries, as well as America, find how difficult it is to secure that excellence in teaching, and that high degree of attainment among physicians and medical students, which all commend and which so few seem to reach. Let us hope that the formation of the section, to which the article alludes, has been one step forward, and in the right direction, and that it may be followed by others which shall ultimately mark an advance.—[EDITOR.]

